# Use of generic medicines by the Brazilian population: an evaluation of PNAUM 2014

**DOI:** 10.1590/S1518-8787.2016050006120

**Published:** 2016-12-01

**Authors:** Andréa Dâmaso Bertoldi, Paulo Sergio Dourado Arrais, Noemia Urruth Leão Tavares, Luiz Roberto Ramos, Vera Lucia Luiza, Sotero Serrate Mengue, Tatiane da Silva Dal-Pizzol, Mareni Rocha Farias, Maria Auxiliadora Oliveira

**Affiliations:** I Departamento de Medicina Social. Faculdade de Medicina. Universidade Federal de Pelotas. Pelotas, RS, Brasil; II Departamento de Farmácia. Faculdade de Farmácia, Odontologia e Enfermagem. Universidade Federal do Ceará. Fortaleza, CE, Brasil; III Departamento de Farmácia. Faculdade de Ciências da Saúde. Universidade de Brasília. Brasília, DF, Brasil; IV Departamento de Medicina Preventiva. Escola Paulista de Medicina. Universidade Federal de São Paulo. São Paulo, SP, Brasil; V Departamento de Política de Medicamentos e Assistência Farmacêutica. Escola Nacional de Saúde Pública Sérgio Arouca. Fundação Oswaldo Cruz. Rio de Janeiro, RJ, Brasil; VI Programa de Pós-Graduação em Epidemiologia. Faculdade de Medicina. Universidade Federal do Rio Grande do Sul. Porto Alegre, RS, Brasil; VII Departamento de Produção e Controle de Medicamentos. Faculdade de Farmácia. Universidade Federal do Rio Grande do Sul. Porto Alegre, RS, Brasil; VIII Departamento de Ciências Farmacêuticas. Centro de Ciências da Saúde. Universidade Federal de Santa Catarina. Florianópolis, SC, Brasil

**Keywords:** Drugs, generic, Drug Utilization, Socioeconomic Factors, Health Surveys

## Abstract

**OBJECTIVE:**

To analyze the existence of differences in the use of generic medicines in Brazil according to demographic and socioeconomic variables and acquisition sources of the medicines.

**METHODS:**

Population-based cross-sectional study, conducted with data from the *Pesquisa Nacional de Acesso, Utilização e Promoção do Uso Racional de Medicamentos* (PNAUM – National Survey on Access, Use and Promotion of Rational Use of Medicines). Data collection took place between September, 2013 and February, 2014 in homes of Brazilian cities (urban area). The use of medicines has been investigated in relation to the treatment of chronic diseases and, in the case of acute events, regarding use over the previous 15 days. Generics were identified by visualization of packaging presented by the users of the medicines. The independent variables used were sex, age, education level, economic class, and region of the Country. The statistical significance of differences between the groups was evaluated by Pearson’s Chi-squared test, considering a 5% significance level.

**RESULTS:**

The prevalence of generic medicines use was 45.5% (95%CI 43.7–47.3). There was no difference considering education level. The prevalence was higher in females (47.0%; 95%CI 44.9–49.0) than in males (43.1%; 95%CI 40.5–45.8), and were higher with increasing age. Generic medicines were more used in the economic class C (47.0%; 95%CI 44.9–49.1) and in the South (50.6%; 95%CI 46.6–54.6) and Southeast (49.9%; 95%CI 46.8–53.0) regions. Generics accounted for 37.3% of the medicines provided by the Brazilian Unified Health System.

**CONCLUSIONS:**

Currently, there is a choice of purchase or free provision by the Brazilian Unified Health System, characterized by quality assurance and reduced price regarding branded medicines considered as reference. In the private market, a considerable part of the population is choosing generic medicines thanks to the availability of this option for virtually all medicines most used by the population.

## RESUMO

**OBJETIVO:**

Analisar se há diferença no uso de medicamentos genéricos no Brasil segundo variáveis demográficas, socioeconômicas e fontes de obtenção dos medicamentos.

**MÉTODOS:**

Estudo transversal de base populacional, conduzido com dados da Pesquisa Nacional de Acesso, Utilização e Promoção do Uso Racional de Medicamentos (PNAUM), com coleta de dados entre setembro de 2013 e fevereiro de 2014 em residências de municípios brasileiros urbanos. O uso dos medicamentos foi investigado em relação ao tratamento de doenças crônicas e, no caso de eventos agudos, quanto ao uso nos últimos 15 dias. Os genéricos foram identificados por visualização das embalagens apresentadas pelos usuários dos medicamentos. As variáveis independentes utilizadas foram sexo, idade, escolaridade, classe econômica e região do País. A avaliação da significância estatística das diferenças entre os grupos foi analisada pelo teste Qui-quadrado de Pearson, considerando nível de significância de 5%.

**RESULTADOS:**

A prevalência de uso de genéricos foi de 45,5% (IC95% 43,7–47,3). Não houve diferença por escolaridade, as prevalências foram maiores no sexo feminino (47,0%; IC95% 44,9–49,0) em relação ao masculino (43,1%; IC95% 40,5–45,8) e foram crescentes com o aumento da idade. Maiores usos de genéricos foram encontrados na classe econômica C (47,0%; IC95% 44,9–49,1) e nas regiões Sul (50,6%; IC95% 46,6-54,6) e Sudeste (49,9%; IC95% 46,8–53,0). Observou-se ainda que os genéricos representaram 37,3% dos medicamentos disponibilizados pelo Sistema Único de Saúde .

**CONCLUSÕES:**

Pode-se concluir que hoje existe uma alternativa de compra ou fornecimento gratuito pelo Sistema Único de Saúde, caracterizada por garantia de qualidade e preço reduzido em relação aos medicamentos de marca comercial considerados como referência. No mercado privado, boa parte da população está optando pelo uso de medicamentos genéricos, graças à disponibilidade dessa opção para praticamente todos os medicamentos mais utilizados pela população.

## INTRODUCTION

The debate on the policy formulation of generic medicines in Brazil has emerged in the early 1990s, during the process of implementation of the Brazilian Unified Health System (SUS). Being a public health system that aims to ensure integral, universal, and free access to health-care for the entire population of the Country, it included, among its activities, the provision of integral therapeutic assistance^a^. In 1993, in the context of health policies in the pharmaceutical area of the Brazilian Ministry of Health (MS) and as a strategy to reduce prices, the Decree 793^b^ was published, presenting advances such as the highlight to generic names on packs; prescription by the generic name; mandatory presence of the pharmacist in the pharmacy; and permission to fraction the packaging.

In 1998, the National Drug Policy (PNM) was released^c^. In addition to establishing guidelines such as the organization of the health surveillance activities and the reorientation of pharmaceutical services, the PNM had among its objectives to promote the use of generic medicines. In 1999, the National Agency of Sanitary Vigilance (ANVISA) (www.anvisa.gov.br) was created, and the Generic Medicines Policy (Law 9,787/99) was instituted^d^.

Until then, the Brazilian pharmaceutical market was composed by two categories of medicines: the “innovators”, today known as reference medicines, which, in addition to their own brand, are registered upon proof of efficacy and safety; and the “similar” drugs, containing the same active ingredients, the same concentration, dosage form, route of administration, dosage, and therapeutic indication of the reference medicine product registered at ANVISA^e^.

From 1999 on, a new category, the generic medicines, entered the national pharmaceutical market. These medicines, in addition to presenting the characteristics of the similar drugs, must prove that they are interchangeable with the reference medicines registered at ANVISA. Therefore, they must present tests that show pharmaceutical equivalence, and, if necessary, bioequivalence (or relative bioavailability) regarding the reference product^16^.

The implementation of the generic policy fulfilled its initial objectives of encouraging commercial competition, improving the quality of medicines, and facilitating the access of the population to medicines treatment^16^. Besides, the national industry became strong, with significant increase in the number of national companies (90.0% of the generic-producing industries), and also of international companies installed in the Country, in addition to a increase of supply and variety of products. In May 2014, the participation of generic medicines on the market amounted to 28.0% of sales^f^.

In 2014, it was already possible to treat most of the more prevalent diseases in the Country with generic medicines, which cost up to 35.0% less than the reference products, thereby improving the affordability of the general population to medicines^f^.

In this article, we analyzed the existence of differences in the use of generic medicines in Brazil according to demographic and socioeconomic variables and acquisition sources of the medicines.

## METHODS

The Brazilian Ministry of Health, to better know the range of the public policies related to access and rational use of medicines in Brazil, held, in partnership with Brazilian universities and research institutes, a national household survey, called *Pesquisa Nacional sobre Acesso, Utilização e Promoção do Uso Racional de Medicamentos* (PNAUM – National Survey on Access, Use and Promotion of Rational Use of Medicines).

The PNAUM, a population-based cross-sectional study, was conducted between September 2013 and February 2014. The interviews were conducted in the households and the data recorded in tablets with a software developed specifically for the application of the questionnaires. The population under study was of individuals of all ages living in private permanent homes in the urban area of the Brazilian territory. The sample size estimates considered eight demographic domains (different sex and age groups) that were replicated to every major Brazilian geographical region, resulting in 40 sample domains and sample size of 38,400 interviews. The selection of the sample was made in three stages: city (primary unity), censitary sector, and household, and the selection of individuals within households was based on the expected proportion of each age and sex group to compose the final sample. The sampling process was complex and resulted in representative sample of Brazil and its five geographical regions, stratified by sex and age groups. Details about the sampling and logistics of data collection can be found in the methodological article of PNAUM^13^.

The research instrument consisted of a set of questionnaires, developed by researchers from Brazilian universities (researchers and their institutions, as well as the complete research instruments, can be found at http://www.ufrgs.br/pnaum). The use of medicines was investigated from the information on previous diagnosis and medical indication for use of medicines for the treatment of specific chronic diseases (hypertension, diabetes, heart diseases, high cholesterol, history of stroke, chronic lung diseases, arthritis, arthrosis or rheumatism, depression). In the case of acute events, question were made regarding the use of medicines in the last 15 days for a series of symptoms or acute health problems and for any other health reason, in addition to the specified ones (infection, sleep medicines, to the nerves, to stomach or bowel problems, pain, fever, flu, cold or allergic rhinitis, vitamin, mineral supplement, appetite stimulant, or tonic). A separated questionnaire was developed for contraceptives, because of the characteristics of this group of medicines, which is unrelated to diseases.

During the interview, it was requested that the respondent showed all the “remedies” in use. A remedy could be a compounding or industrialized medicine (sold in pharmacies and prescribed by physicians), as well as teas, homeopathic products, and medicinal plants, for example.

The team was trained to recognize generic medicines by observing their packaging and blisters. It was considered as packing the box of the medicine, pack, envelope, tube, bottle, or other container that provided information about the medicine.

This article analyses were carried out using two databases with different denominators. To estimate the prevalence of use of generic medicines, the database containing data of the people included in the sample was used. The prevalence of use of generic medicines was estimated considering in the numerator those who used at least one generic medicine and in the denominator those who presented at least one packing of the medicines used. The prevalence and 95% confidence intervals (95%CI) were also estimated according to the individual characteristics of the respondent: sex (male; female); age in full years (0-9; 10-19; 20-59; 60 or more); education level in complete years (0-8; 9-11; 12 or more); economic classification (A/B; C; D; E) according to the Brazilian Economic Classification Criterion developed by the *Associação Brasileira de Empresas de Pesquisa* (CCEB 2013/ABEP – http://www.abep.org/); and geographical region of residence (North; Northeast; Southeast; South; Midwest).

The sources for obtaining the generic medicines (pharmacy of SUS – public network, private pharmacy, pharmacy from the *Farmácia Popular* program [Popular Pharmacy Program], and other sources) and the reason for the use of generics (to treat chronic diseases or for any eventual or acute diseases), in the total sample and by age groups, were analyzed from the database of medicines. We used as denominator all medicines whose packaging were presented to the interviewer in favorable conditions to allow the verification of the characteristics to classify them as generics or not (n = 37,419). Specifically to contraceptives, information about being generics were not collected from the characteristics of the packaging presented. Therefore, this group of drugs was not analyzed.

The analyses were carried out in the statistical package Stata 12.0 (StataCorp LP, College Station, Texas, USA), using the appropriate svy commands for the analysis of complex samples and ensuring the necessary weighting (post-stratification weights to correct the response rate bias). The estimated percentages were weighted to adjust the demographic distribution of the PNAUM sample to the distribution of the Brazilian population.

The medicines were classified according to levels 1 and 2 of the Anatomical Therapeutic Chemical (ATC) classification system^20^. The highest frequencies were analyzed on the level 2 of the ATC classification according to the most frequent occurrences at level 1 and, for each one of them, the proportion of generics was estimated regarding the total medicines reported in the respective groups. Medicines that do not have a generic presentation on the market may be included in this analyses, which is reflected in the percentages of use of this type of medicines.

The statistical significance of differences between the groups was evaluated by Pearson’s Chi-squared test, considering a 5% significance level.

The study was approved by *Comissão Nacional de Ética em Pesquisa* (CONEP – Brazilian National Commission for Ethics in Research; Opinion 398,131, of September 16, 2013) and all the interviews were conducted after the respondents or their legal guardians (in case of children under 18 or people unable to answer their own questionnaire) read and signed the informed consent form.

## RESULTS

The total study sample included 41,433 individuals with distribution by sex and age compatible with the Brazilian population according to the 2010 Census (percentage weighted by sampling weights). The response rates of households were around 50.0%, including as losses the unvisited households. Individuals’ response rates were around 90.0%. The methodological article presents a detailed table of response rates by sex and age groups and by geographic regions^13^.

The prevalence of use of at least one generic medicine, estimated among individuals who presented at least one package of medicines (n = 16,316), was of 45.5% (95%CI 43.7–47.3). We did not observe any differences by education level. However, the prevalence was greater in females than in males (47.0% *versus* 43.1%, respectively), and was higher with increasing age, with the group aged 60 years old or more using 1.7 times more generics than the group from zero to nine years old. The economic class C showed higher prevalence of generic medicines use (47.0%; 95%CI 44.9–49.1), as well as individuals residing in the South (50.6%; 95%CI 46.6–54.6) and Southeast (49.9%; 95%CI 46.8–53.0) regions (Table 1).

**Table 1:** Prevalence of use of at least one generic medicine according to demographic and socioeconomic characteristics. PNAUM, Brazil, 2014.

Variable	Prevalence of use of generics^a^		

	%^b^	95%CI	p
Sex			0.013
Male	43.1	40.5–45.8	
Female	47.0	44.9–49.0	
Age (years)			< 0.001
0-9	33.5	29.1–37.8	
10-19	33.0	26.8–39.2	
20-59	43.6	41.5–45.7	
≥ 60	56.2	54.1–58.3	
Education level^c^			0.819
0-8 years	45.5	43.4–47.6	
9-11 years	44.4	40.4–48.3	
≥ 12 years	45.9	43.1–48.6	
Economic classification^d^			0.040
A/B	42.6	39.5–45.7	
C	47.0	44.9–49.1	
D/E	44.6	41.3–48.0	
Region			< 0.001
North	32.7	28.5–37.0	
Northeast	37.9	35.8–40.0	
Southeast	49.9	46.8–53.0	
South	50.6	46.6–54.6	
Midwest	45.1	41.9–48.4	

Total	45.5	43.7–47.3	-

^a^ Only medicines whose packages were shown in the interview allowed the classification as a generic, which corresponds to 63.2% of the medicines. The prevalence was calculated among those who presented at least one package of their medicines (n = 16,316 people).

^b^ Percentages adjusted by sample weights and post-stratification according to age and sex.

^c^ The education level variable presents 278 missings.

^d^ The economic classification variable according to the Brazilian Economic Classification Criterion 2013 of the *Associação Brasileira de Empresas de Pesquisa* (http://www.abep.org) presents 77 missings.

**Figure 1:**
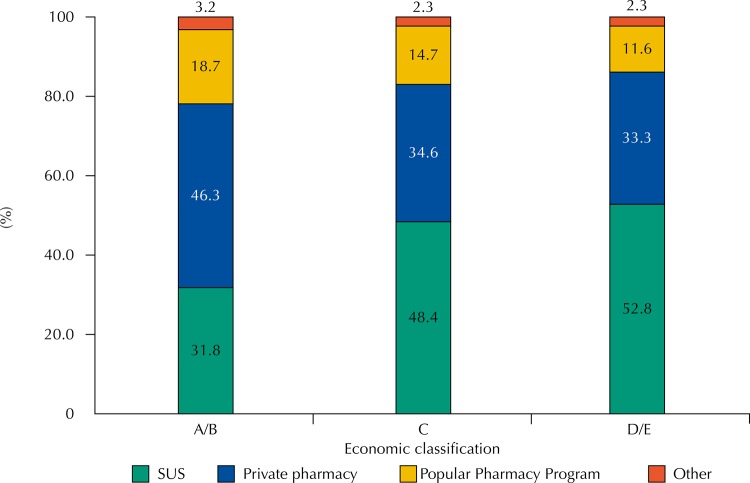
Sources for obtaining generic medicinesa, stratified by economic classificationb of the users of these medicines. PNAUM, Brazil, 2014. (N = 10,870 medicinesc)

Packaging or blisters of 37,419 medicines (63.2% of the total medicines used) were presented at the time of the interviews, allowing the visualization of the characteristics that differentiate generics from other medicines. Of these, 31.2% (95%CI 29.8–32.6) were classified as generics. Regarding the sources for obtaining them, the classes with higher purchasing power (A/B) obtained the generic medicines, mainly, in private pharmacies (46.3%), while in classes C and D/E (less spending power), about 50.0% of generics were obtained in pharmacies from the public network (SUS) (Figure 1). We also observed (data not shown in the figure) that generics accounted for 37.3% of the medicines obtained by SUS, 53.7% of those provided by the Popular Pharmacy Program, 24.2% in private pharmacies, and 16.6% from other sources (charity institution or churches, free samples, friends, relatives, or neighbors).

SUS: Brazilian Unified Health System

^a^ Only medicines whose packages were shown in the interview allowed the classification as a generic, which corresponds to 63.2% of the medicines. Percentages adjusted by sample weights and post-stratification according to age and sex*.*

^b^ The economic classification variable according to the Brazilian Economic Classification Criterion 2013 of the *Associação Brasileira de Empresas de Pesquisa* (http://www.abep.org) presents 77 missings.

^c^ Number of medicines classified as generics that have information on economic classification and acquisition source.

**Figure 2:**
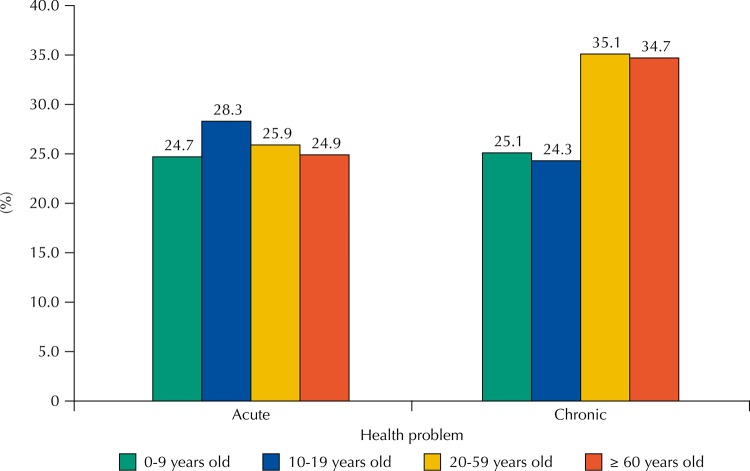
Type of health problem treated with generic medicinesa according to the age of the users. PNAUM, Brazil, 2014. (N = 11,215 medicinesb)

^a^ Only medicines whose packages were shown in the interview allowed the classification as a generic, which corresponds to 63.2% of the medicines. Percentages adjusted by sample weights and post-stratification according to age and sex.

^b^ Number of medicines classified as generics that have information on age and type of health problem.

Considering the totality of generics used by the individuals from the sample, 68.8% were used in the treatment of chronic diseases and 31.2% in acute or potential health problems. The use of generics to treat acute health problems was similar among the age groups. Regarding the generics used for chronic diseases, in the younger groups (children and adolescents), the proportion of generics was equivalent to the medicines used to treat acute problems. However, in adults and older adults, the proportions of generics were superior, reaching approximately 40.0% higher in older people regarding children (Figure 2).

Table 2 presents the most used therapeutic groups and sub-groups in the total sample of the packagings presented. The frequency distribution of these groups is comparable to the sample that includes all medicines (regardless of the presentation of the package) (data not presented). Medicines for the cardiovascular system (35.9%), nervous system (18.6%), and alimentary tract and metabolism (16.0%) were the most used ones. Within these therapeutic groups, generics accounted for 40.1%, 35.3%, and 29.5%, respectively, of the medicines used. Regarding therapeutic subgroups, the highest proportions of generics occurred for agents acting on the renin-angiotensin system, psychoanaleptics, and drugs used for diabetes (49.2%, 40.9%, and 41.3%, respectively). In the ATC2 subgroup of vitamins (A11), which represented 12.2% of the medicines for the alimentary tract and metabolism analyzed, no generic was used, because there was no generic on the market for this type of medicine at the time of the research.

**Table 2:** Therapeutic groups and subgroups most used in the total sample considering only the medicines that had their packages or blisters presented, and proportion of generics in the total of each group. PNAUM, Brazil, 2014. (N = 37,419)a

ATC classification^b^ level 1 and level 2	Most used therapeutic groups	

	All (%)^c^	Generics (%)
C: Cardiovascular system	**35.9**	**40.1**
C09 Agents acting on the renin-angiotensin system	36.9	49.2
C03 Diuretics	22.0	37.4
C07 Beta-blockers	14.1	43.0
C10 Lipids modifying agents	13.4	33.9
N: Nervous system	**18.6**	**35.3**
N02 Analgesics	40.8	37.8
N06 Psychoanaleptics	19.3	40.9
N03 Antiepileptics	17.7	27.2
N05 Psycholeptics	17.3	36.7
A: Alimentary tract and metabolism	**16.0**	**29.5**
A10 Medicines used for diabetes	34.2	41.3
A02 Medicines for acid-related disorders	31.6	40.7
A11 Vitamins	12.2	0
A03 Medicines for functional gastrointestinal disorders	9.3	18.6
M: Musculoskeletal system	**8.7**	**20.5**
M01 Anti-inflammatory and antirheumatic medicines	63.1	25.3
M03 Muscle relaxants	25.6	11.3
M05 Medicines for treatment of bone diseases	8.4	7.3
R: Respiratory system	**5.1**	**18.8**
R03 Agents against airway obstructive diseases	29.9	13.4
R06 Antihistamines for systemic use	29.3	30.5
R05 Cough and cold preparations	21.9	23.4
R01 Preparations for nasal use	18.6	3.7
J: Anti-infectives for systemic use	**2.7**	**37.2**
J01 Antibacterials for systemic use	88.7	40.0
J05 Antivirals for systemic use	6.8	20.8
J02 Antifungal agents for systemic use	2.4	3.4
H: Systemic hormonal preparations, excluding sex hormones and insulins	**2.3**	**27.8**
H03 Thyroid therapy	64.0	23.2
H02 Corticosteroids for systemic use	35.8	36.4
B: Blood and haematopoietic organs	**2.0**	**15.4**
B03 Anti-anemic preparations	57.5	6.3
B01 Antithrombotic agents	41.6	27.9
Other ATC level 1	**8.6**	-
Total ATC level 1 groups	**100**	**31.2**

^a^ Only medicines whose packages were shown in the interview allowed the classification as a generic, which corresponds to 63.2% of the medicines. Contraceptives, despite their packaging, were not classified as generics, therefore, they are not part of this analysis.

^b^ Anatomical Therapeutic Chemical (ATC) classification system. Only the most frequent ATC level 2 are presented for each ATC1 group.

^c^ Percentages adjusted by sample weights and post-stratification according to age and sex.

The values in bold refer to the total from the level 1 of ATC classification.

Table 3 lists the medicines, in order of frequency of use, including about 50.0% of the total sample (first column of the table). The second column indicates the percentage of presentation of packaging for each of these medicines, allowing to visualize how much of the drugs was assessed regarding the proportion of generic indicated in the third column. The largest percentages of presentation of packaging (above 80.0%) were found for medicines used for treatment of chronic diseases, and the lowest (less than 50.0%), for those used to treat signs, symptoms, or acute affections. The percentages of generics among the most commonly used medicines that could be analyzed ranged from 5.2% (caffeine + orphenadrine + dipyrone) to 66.4% (losartan).

**Table 3:** List of the most used drugsa and percentage of use of generics estimated among the medicines that had their packaging presented. PNAUM, Brazil, 2014.

Drugs	% of use over the total number of drugs	% of packaging presentation	% of generics in the sample^b^
		
	(n = 57,424)	(n = 57,424)	(n = 37,419)
Dipyrone	4.6	42.1	43.8
Hydrochlorothiazide	4.1	85.1	37.9
Paracetamol	4.0	40.9	55.4
Losartan	3.6	84.3	66.4
Omeprazole	3.4	66.1	40.9
Simvastatin	2.9	66.8	34.3
Ethinyl estradiol + levonorgestrel^c^	2.7	63.2	-
Caffeine + orphenadrine + dipyrone	2.6	33.2	5.2
Metformin	2.5	79.2	52.3
Captopril	2.3	78.2	41.9
Enalapril	2.3	82.2	39.5
Atenolol	1.9	77.9	53.6
Ibuprofen	1.6	66.4	30.0
Acetylsalicylic acid	1.6	72.7	17.2
Diclofenac	1.5	63.1	27.3
Clonazepam	1.3	81.7	39.8
Levothyroxine	1.3	80.3	24.1
Amlodipine	1.2	82.4	24.3
Amoxicillin	1.1	50.4	46.4
Caffeine + carisoprodol + diclofenac + paracetamol^d^	1.1	59.0	-
Glibenclamide	1.0	83.4	34.1

^a^ Corresponding to approximately 50.0% of all medicines used by the sample.

^b^ Only medicines whose packages were shown in the interview allowed the classification as a generic, which corresponds to 63.2% of the medicines. Percentages adjusted by sample weights and post-stratification according to age and sex.

^c^ Contraceptives were not classified as generics.

^d^ Drug that does not have a generic version on the market.

## DISCUSSION

The generic medicines policy^d^ is part of the PNM^c^, since it promotes access by stimulating competition and reduction of prices and spending with medicines. The evaluation of this policy includes studies that analyze the question of prices or that describe the pattern of use of generics. There are many examples of reduction of prices of medicines as a result of the entry of generics in the pharmaceutical market. In this research, the focus of the analyses was to show the use of this type of medicines in the national territory.

Studies indicate, especially at the beginning of the implementation of policies related to generics, some resistance to replace medicines with established brands in the market for generics. However, with time, this scenario has changed because of the benefits arising from lower price, as well as for the guarantee that these medicines are interchangeable with those of reference, by the requirement of proof of pharmaceutical equivalence and bioavailability.

The fact is that, in Brazil, after little more than 10 years from the entry of generics on the market, we have seen that the prevalence of use of this group reached 45.5% of the population – much higher than the prevalence found in previous researches, which have obtained prevalences of less than 10.0%. Approximately 1/3 of all medicines used were generic, showing the important participation of this group of medicines on the market. Generics are present in over 30.0% of the pharmacological groups of great demand, such as medicines for the cardiovascular system, nervous system, and in some subgroups, such as medicines for diabetes, disturbances related to acidity of the alimentary tract, antihistamines, antibacterials, and corticosteroids of systemic use.

Analyzing the most widely used medicines in the population, it is possible to estimate the participation of generics among the total of medicines used. The most used ones in the generic version were for treating chronic diseases (losartan, metformin, atenolol), but we also observed a medication of high prevalence for acute health problems (paracetamol) and one antimicrobial (amoxicillin). It is important to note the low percentages of generics found for the treatment of bone diseases, airways obstructive diseases, and anti-anemic preparations.

Regarding the characteristics of users of generics, no difference was found regarding education level. Studies of overall use of medicines also found no difference for education level after adjusted analysis. Concerning the differences by sex, invariably, the studies showed higher prevalence of global use of medicines among women. In fact, in general, women use more medicines than men, especially from adolescence on, regardless of the inclusion of contraceptives in the analysis. This suggests that the choice of generic medicines is not related to sex. The higher prevalence of use of generics in females can be a simple reflection of the greater use of medicines in this group.

The use of generics was directly proportional to age, which is consistent with the literature on global use of medicines. Apparently, the preference for using generics occurs between adults and older adults for treatment of chronic diseases. The proportion of generics used for chronic diseases of children and adolescents may be smaller because of the profile of chronic diseases in these age groups, in which it is common for individuals to suffer from, at most, one chronic condition, as opposed to what occurs with adults and, especially, older adults.

Regarding use according to economic classes, the groups of greatest spending power (A/B) presented less frequent use, contrary to what is observed in relation to the use of all medicines in general. Economic classification is directly related to the use of any medicine. However, considering specifically generics, it is possible that groups of greater spending power will choose known brands rather than cheaper options, which, in this case, would be the generic version of these medicines. Classes A/B get their generics, primarily, in private pharmacies, and classes C and D/E, primarily in SUS, but this procedure is expected for any type of medicine.

The richest geographic regions (South and Southeast) used more generics than the poorest ones (North and Northeast). For being a cheaper medicine, we expected the opposite. However, similar medicines can be even cheaper than generics, and the option for this type of medicine may have influenced this result. The lack of studies at the time of PNAUM data collection comparing prices of generic medicines with others prevents conclusions on the subject to be more decisive. However, we raised the issue of price as a possibility as well as we should consider the possibility of lower availability of generics in the North and Northeast regions^14^. The Southeast region concentrates most of the generics market in the Country and there is a direct correlation between the number of pharmacies in each Brazilian region and sales of generics^g^. The North and Northeast regions have the lowest proportion of number of pharmacies by population, which may indicate lower offer of this type of medicine.

This study presents national and regional representation, which allowed, for the first time, an overview of the use of generic medicines, focus of important government policy aimed at increasing access to medicines. On the other hand, it also presents some limitations, inherent to an investigation of such magnitude. The final response rates included, in addition to the unvisited households, vacant households. This methodological approach may overestimate non-response rates. In addition, there may be some level of commitment of the data arising from the loss of households from the original sample, considering that the lost households, on average, were richer. A detailed discussion of this limitation can be found in the PNAUM methodological article^13^. Since the information depend on the respondents’ statements, a certain degree of recall error is possible when reporting the medicines used (limitation of the type of data collection, which may lead to an underestimation of the prevalence of use of medicines). Also, because it was not possible to fully obtain the packages of medicines, it was not possible to analyze all drugs used regarding packaging or blisters characteristics, which would enable to identify whether the product used was a generic or not. With this, only 63.0% of medicines could be classified. However, we observed that the distribution of pharmacological groups and most used drugs was equivalent between medicines whose packaging were submitted and the others. Specifically in the case of contraceptives, for a methodological issue, the use of generics was not analyzed.

With this overview of the generic medicines use in Brazil, we can conclude that today there is a choice of purchase or free provision by the Brazilian Unified Health System, characterized by quality assurance and reduced price regarding medicines of market brands considered as reference. In the private market, much of the population who can exercise their power of choice is choosing this type of medicine, made possible by the existence of a generic option for virtually all the most used medicines by the population.

## Footnotes

a Brasil. Artigo 6°, de 5 de outubro de 1988. São direitos sociais a educação, a saúde, a alimentação, o trabalho, a moradia, o transporte, o lazer, a segurança, a previdência social, a proteção à maternidade e à infância, a assistência aos desamparados, na forma desta Constituição. In: Brasil. Constituição (1988). Brasília (DF): Senado; 1988.

b Brasil. Decreto n° 793, de 5 de abril de 1993. Altera os Decretos nºs 74.170, de 10 de junho de 1974 e 79.094, de 5 de janeiro de 1977, que regulamentam, respectivamente, as Leis nºs 5.991, de 17 de janeiro de 1973, e 6.360, de 23 de setembro de 1976, e dá outras providências. *Diario Oficial Uniao*. 6 abr 1993.

c Ministério da Saúde. Portaria n° 3.916, 30 de outubro de 1988. Aprova a Política Nacional de Medicamentos. *Diario Oficial Uniao*. 10 nov 1988;Seção 1:18-22.

d Brasil. Lei n° 9.787, de 10 de fevereiro de 1999. Altera a Lei no 6.360, de 23 de setembro de 1976, que dispõe sobre a vigilância sanitária, estabelece o medicamento genérico, dispõe sobre a utilização de nomes genéricos em produtos farmacêuticos e dá outras providências. *Diario Oficial Uniao*. 11 fev 1999.

e Brasil. Decreto n° 3.961, de 10 de outubro de 2001. Altera o Decreto no 79.094, de 5 de janeiro de 1977, que regulamenta a Lei n 6.360, de 23 de setembro de 1976. *Diario Oficial Uniao*. 11 out 2001;Seção 1:15.

f Associação Brasileira das Indústrias de Medicamentos Genéricos. Pro-genéricos. São Paulo (SP): Associação Brasileira das Indústrias de Medicamentos Genéricos; 2014 [cited 29 Nov 2015]. Available from: http://www.progenericos.org.br/

g Novaretti MCZ, Quitério LM, Piscopo MR. Desafios na gestão de medicamentos genéricos no Brasil: da produção ao mercado consumidor. In: Anais do 38º Encontro da ANPAD; 13-17 set 2014 [cited 29 Nov 2015]. Rio de Janeiro (RJ). Available from: http://www.anpad.org.br/admin/pdf/2014_EnANPAD_GOL512.pdf
